# Oral health management in children with severe congenital neutropenia with periodontitis: Case report

**DOI:** 10.1097/MD.0000000000039086

**Published:** 2024-07-26

**Authors:** Si-Yu Tao, Min Yao, Yu-Lei Dong, Xue-Jing Lin, Diwas Sunchuri, Zhu-Ling Guo

**Affiliations:** a School of Dentistry, Hainan Medical University, Haikou, PR China; b Children’s Hospital, Nanjing Medical University, Nanjing, PR China; c School of International Education, Hainan Medical University, Haikou, PR China; d Department of Health Management Center, The First Affiliated Hospital of Hainan Medical University, Haikou, PR China.

**Keywords:** allogeneic hematopoietic stem cell transplantation, congenital neutropenia, oral health management, periodontitis

## Abstract

**Rationale:**

Severe congenital neutropenia (SCN) is a rare and heterogeneous genetic disease. By describing the diagnosis and treatment of a child with SCN and periodontitis, this case provides a reference for the oral health management of a child with SCN and periodontitis.

**Patient concerns:**

We describe a boy with clinical manifestations of oral bleeding, neutropenia, recurrent fever, and other recurrent infections. The absolute neutrophil count (ANC) was <0.50 × 10^9^/L most of the time. Morphological examination of bone marrow cells showed active granulocyte hyperplasia and dysmaturation.

**Diagnoses:**

According to the clinical manifestations, hematological examination and gene detection results, the child was diagnosed as SCN with chronic periodontitis.

**Interventions:**

Periodontal treatment was performed after informed consent was obtained from the child guardian. These included supragingival and subgingival cleaning, hydrogen peroxide and saline irrigation, placement of iodoglycerin in the gingival sulcus, and oral hygiene instruction. Hematopoietic stem cell transplantation (HSCT) was performed later.

**Outcomes:**

One month after initial periodontal treatment, oral hygiene was well maintained and gingival swelling had subsided. Probing depth (PD) index on periodontal probing and bleeding was significantly reduced. However, there was no significant change in blood routine and other indicators before and after periodontal treatment.

**Conclusion:**

Once SCN is diagnosed, individualized treatment plans can be developed according to the characteristics of the disease and its impact on oral health, which can effectively control the interaction between SCN and periodontal disease and reduce the occurrence of serious infection.

## 1. Introduction

Neutropenia is a group of syndromes in which the absolute neutrophil count (ANC) in peripheral blood is lower than 1.5 × 10^9^/L. Severe congenital neutropenia (SCN) is a heterogeneous group of inherited bone marrow failure syndromes. SCN was first reported by Kostmann, a Swedish pediatrician, in 1956, also known as Kostmann syndrome. It is mainly characterized by the reduction of ANC in peripheral blood and the arrest of granulocyte production in bone marrow at the promyelocytic/myelocytic stage. It is mainly manifested as a decrease in the absolute count of neutrophils in peripheral blood (<0.5 × 10^9^/L).^[1]^ Neutrophils play an important role in the first line of defense during acute inflammatory responses and host defense against bacterial infections. The lack of these cells leaves the human body vulnerable to life-threatening bacterial infections.^[2–5]^ The clinical symptoms of congenital neutropenia include sepsis, recurrent respiratory tract infections, oral ulcers, chronic gingivitis, bacterial skin infections, and urinary tract infections. In this report, we present the history, clinical, imaging, and hematologic findings of a child with SCN and periodontitis in order to improve our understanding of the disease, and provide reference for the clinical diagnosis, treatment and prognosis of SCN patients with periodontitis.

## 2. Case presentation

**Figure 1.**
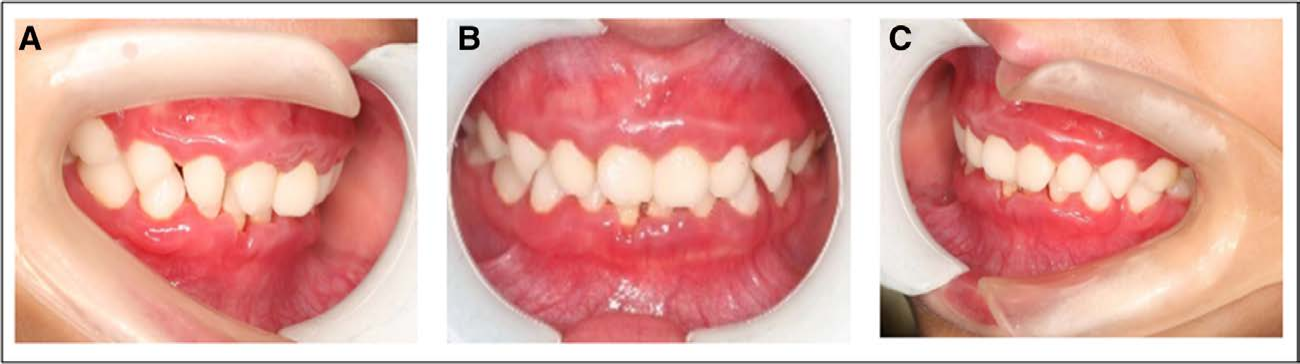
Periodontal condition before treatment. In primary dentition, the gingival papillae of the whole mouth were soft and dark red with obvious swelling. (A) right side view; (B) positive view; (C) left side view.

**Figure 2.**
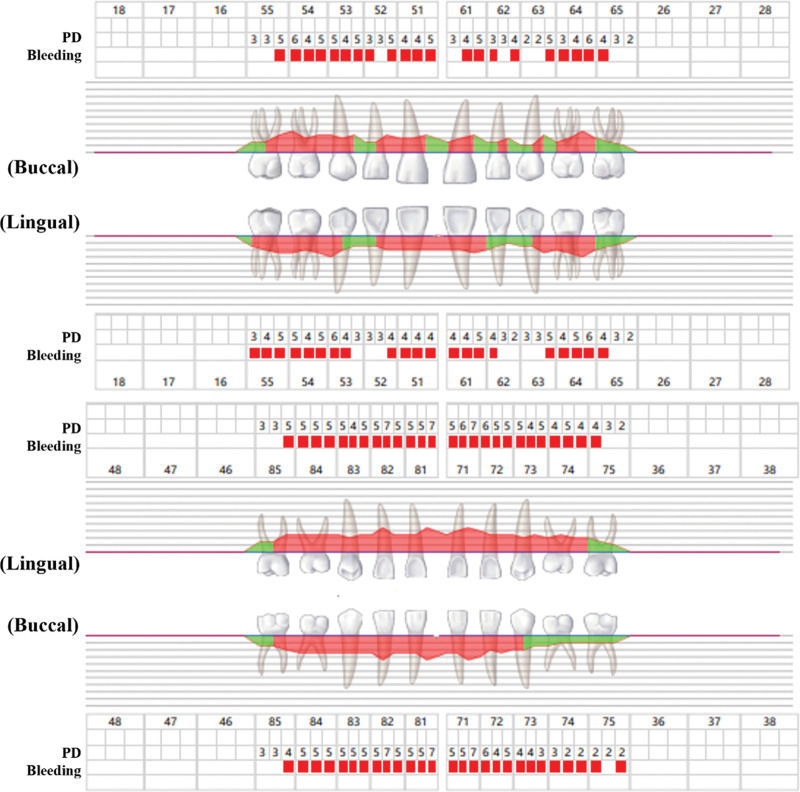
Periodontal probing record at the initial diagnosis. It easily bleeds when probing in the full tooth position. Probing depth (PD) was 2 to 7 mm, and deep periodontal pockets were detected.

**Figure 3.**
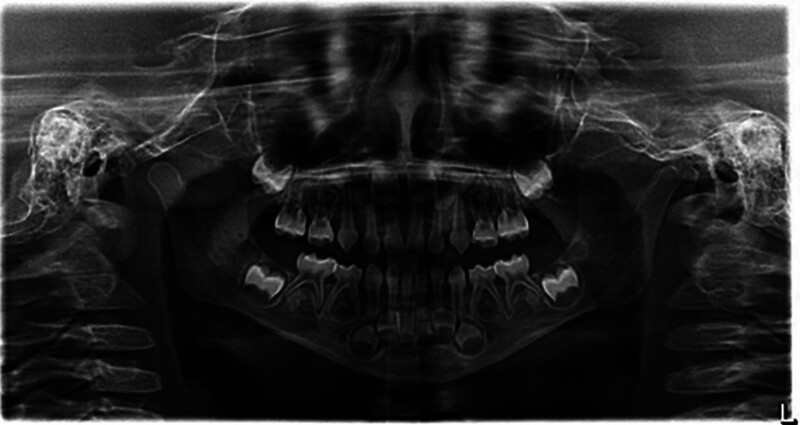
Oral panorama of the child at initial diagnosis. The alveolar bone of the children had different degrees of absorption, and the absorption of the lower anterior teeth was more significant, which was mainly angular resorption.

A 3-year-old male child was referred to the Department of Stomatology, the First Affiliated Hospital of Hainan Medical University by a pediatrician because of red, swollen and bleeding gums throughout the mouth. He had a past history of recurrent cervical lymphadenitis, fever, and cough. Neutrophil count (ANC) ranged from 0.17 × 10^9^/L to 0.22 × 10^9^/L. The patient had received treatment and received a diagnosis of SCN in pediatrics. There were no obvious abnormalities in general development and normal intelligence. The parents were not consanguineous, and there were no similar patients in the family. Informed consent was obtained from the child parents to allow to be described in this report. Maxillofacial and oral examinations revealed bilateral symmetry of the face and palpable submandibular lymph node enlargement in the neck. The oral hygiene was general, the gums were red and swollen throughout the mouth, the gums were prone to bleeding on probing, the probing depth (PD) was 2 to 7 mm, and the deep periodontal pockets could be probed in some teeth (Figs. 1 and 2). Panoramic radiograph showed that the alveolar ridge of the child was absorbed, showing angular absorption (Fig. 3).

Bone marrow smear showed active granulocyte proliferation with maturation disorder, active erythroid proliferation, significantly active megakaryocyte proliferation, and clustered platelets. Target regions of disease-related genes were captured and deeply sequenced in the DNA samples of patients, with an average sequencing depth of 500 to 1000X. Mutation analysis method is the SFT, PolyPhen 2; LRT; MutationTaster. A heterozygous missense mutation in *vwf* gene was found in the child, which was inherited from his father. A mutation was found on chromosome 12 at a specific position of 6127768. In the EXON28 region of NM_000552 gene, the 4816 base changed from A to G, leading to the 1606 amino acid change from methionine to valine in the protein. This amino acid substitution may affect the function of the protein, but further studies are needed to determine the specific effects (Table 1). However, the variant was predicted to be a “benign or suspected benign genetically altered” variant according to the ACMG/AMP criteria. Further clinical examination of patients and not confirmed Von Willebrand disease (VWD). The white-cell count was 1.88 × 10^9^/L, hemoglobin 101 × 10^12^/L, and neutrophil count 0.12 × 10^9^/L (Table 2). According to the clinical manifestations, hematological examination and gene detection results, the child was diagnosed as SCN and chronic periodontitis.

**Table 1:** Genetic screening of *vwf* variants in children.

Item	Result	Item	Result
Chromosome	chr12	Mutation type: Father	Heterozygosis
Location	6127768	Mutation type: Mother	-
Mutation type	Missense mutation	Mutation type: Patient	Heterozygosis
Background frequency	0.000199681	Correlation	3
Amino acid variation NM_000552:EXON28:C.4816A > G:p.M1606V			

**Table 2:** Changes in main indicators of blood routine before and after periodontal treatment.

Item	Before	After	Reference value
White blood cell count	1.88	2.79	3.5–10.0 (10^9^/L)
Neutrophilic granulocyte percentage	6.2	6.12	40.0–75.0 (%)
Percentage of lymphocytes	78.2	81.01	20.0–50.0 (%)
Neutrophil count	0.12	0.17	1.8–6.3 (10^9^/L)
Hemoglobin	101	113.0	110–140 (g/L)
Blood platelet count	271	274.0	125–350 (10^9^/L)
C-reactive protein	8	5.8	0–10.0 (mg/L)

**Figure 4.**
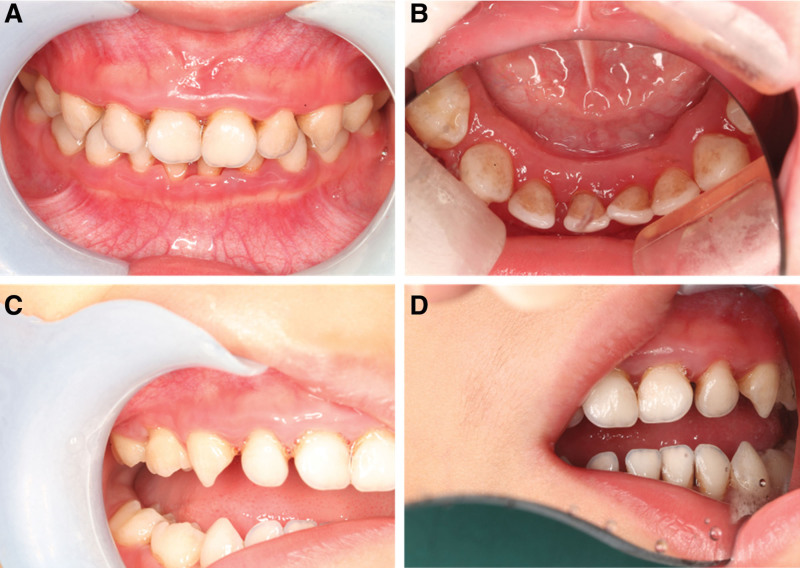
Intra-oral situation of the child 1 month after the end of periodontal treatment. There was no redness, swelling and bleeding in the gums, the swelling of the gums subsided, and the root surface of the teeth was exposed. (A) positive view; (B) mandibular surface view; (C) right side view; (D) left side view.

**Figure 5.**
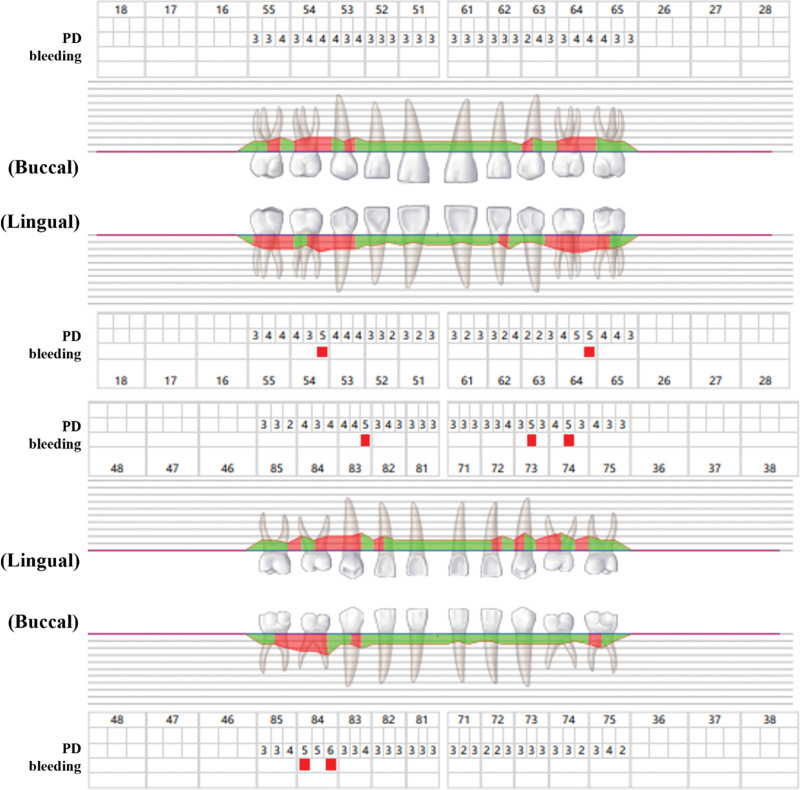
The periodontal probing status was recorded 1 mo after the end of periodontal treatment. The gingival inflammation was significantly reduced. PD values were statistically different (*P* < .05). PD = probing depth.

After considering the patient condition, it was considered that the patient had no obvious coagulation dysfunction. Periodontal treatment was performed after obtaining the informed consent of the children guardians. It included supragingival and subgingival scaling, hydrogen peroxide and normal saline irrigation, and iodine glycerin placement in the gingival sulcus. Patients with SCN are prone to infection due to the low immune function. Therefore, these patients should take oral antibiotics in advance during the treatment to prevent infection. Oral hygiene education was carried out for children and their families, and children were told to strengthen oral hygiene. One month after initial periodontal treatment, oral hygiene was well maintained and gingival swelling had subsided (Fig. 4). PD index on periodontal probing and bleeding were significantly reduced (Fig. 5). However, there was no significant change in blood routine and other indicators before and after periodontal treatment (Table 2). The patient was diagnosed with congenital neutropenia, and no direct significant pathogenic loci were found in related genetic and hematological gene tests, including ELANE and other genes. The patient had recurrent infections since birth, and the infection recurs after symptomatic infusion of granulocyte colony-stimulating factor (G-CSF) and other drugs to increase white blood cells. The Chinese Bone Marrow Bank found 9/10 matched donors, and the parents strongly requested unrelated HSCT. After completing the relevant pre-transplantation examination, there was no adverse indication for transplantation. After completing the relevant examination, the child was treated with bone marrow HSCT after Thi + BFC + ATG regimen, and the prognosis was good. The oral and systemic conditions after BMT remain to be followed up.

## 3. Discussion

SCN is a rare disease with a prevalence of approximately 3 to 8.5 cases per million people.^[6,7]^ Neutrophils are the most abundant white blood cells in the human blood system and play an important role in antimicrobial immunity.^[8]^ Patients with SCN are initially found to have abscesses located in various parts of the body such as the skin, lungs, and mouth. The decrease in the number of neutrophils changes the host defense ability, and the immunity is low, which is prone to repeated serious bacterial and fungal infections, and even life-threatening.^[9]^ This immune disorder in SCN patients is manifested in the oral cavity as the homeostasis disorder of oral commensal microbiota.^[10]^ It has been reported that the saliva of SCN patients has a higher richness of bacterial flora than that of normal controls.^[10]^ Changes in the abundance of oral flora caused by the decrease in neutrophil count make SCN patients susceptible to gingival and periodontal inflammation.^[11]^ Periodontal disease is the response of the gums to dental plaque, which is influenced by systemic and genetic factors.^[12]^ SCN patients complicated with periodontal inflammation can involve primary dentition and permanent dentition, which is typically characterized by rapidly progressive periodontitis, gingival swelling and bleeding, extensive absorption of alveolar bone, tooth loosening, and eventually lead to premature tooth loss.^[13]^ Correspondingly, the rapid progression of these inflammations suggests a severe disturbance of neutrophil release into the peripheral blood.^[14,15]^ In addition, higher concentrations of inflammatory cytokines, such as IL-1β, IL-2, IL-4, EGF and HGF, can be detected in the gingival crevicular fluid of SCN patients.^[10,16]^ This will further aggravate the periodontal inflammatory response. The periodontal damage in the case presented in this article may be related to the low resistance caused by the lack of neutrophils. In this case, the periodontal inflammation was effectively controlled after periodontal intervention and oral hygiene maintenance. Therefore, long-term oral maintenance and regular professional oral health guidance can effectively prevent and treat periodontal infection. Plaque control is a necessary measure to prevent and treat periodontal disease in SCN patients.^[17]^

SCN represents a heterogeneous group of diseases that may be caused by genetic defects or gene mutations.^[18]^ Genetic mutations are found in nearly 60% of SCN patients. ELANE and HAX1 were the most common mutation types. The cases through genetic testing, however, did not see meaningful pathogenic site directly. However, a heterozygous missense mutation in *vwf* gene was found in this case. The *vwf* gene is a von Willebrand factor that is synthesized and secreted by endothelial cells. The *vwf* gene plays an important role in the normal coagulation mechanism. *vwf* gene can bind to collagen fibers and platelets at the same time. When the blood vessel is ruptured, a large number of platelets adhere to collagen fibers with *vwf* as an intermediary protein. Thus, *vwf* is a marker of endothelial injury and dysfunction.^[19]^ Mutations in the *vwf* gene will lead to decreased platelet adhesion, coagulation disorders, and then the body has a tendency to bleeding. VWD is the most common inherent hemorrhagic disorder caused by *vwf* deficiency or dysfunction.^[20]^ Gingival bleeding is a common symptom of VWD. Gingival bleeding is the main symptom of gingivitis caused by dental plaque and untreated periodontal disease. Relevant scholars have shown that the number of supragingival plaque in VWD is positively correlated with gingival inflammation.^[21]^ However, other scholars have suggested that gingival bleeding in VWD patients may be caused by gingival inflammation rather than a real symptom.^[22]^ In this case, there was no uncontrollable bleeding during periodontal treatment, and the blood routine examination showed normal coagulation function. It is speculated that the child still has *vwf* gene mutation but does not show any symptoms. Therefore, it remains to be confirmed by further studies whether the gingival bleeding in this child was due to dental plaque and periodontal disease or a true symptom caused by this hemorrhagic disease. Further cases and studies are also needed to establish a clear causal relationship between the *vwf* gene and SCN and periodontitis.

Many drugs are recommended for the treatment of SCN, such as G-CSF, corticosteroids, androgens, prophylactic antibiotics or cytotoxic therapies.^[23,24]^ The use of G-CSF is primarily used in patients with myelopoiesis defects and has been shown to be a life-saving intervention in patients with SCN. Treatment of SCN patients with G-CSF improves peripheral neutrophil counts and reduces the risk of infection.^[25]^ However, long-term use of recombinant G-CSF significantly increases the risk of transformation of CN patients to secondary myelodysplastic dyndrome (MDS) or acute myeloid leukemia (AML).^[26]^ Hematopoietic stem cells (HSC) transplantation to treat many blood disease, acute myeloid leukemia, acute lymphoblastic leukemia, myelodysplastic syndrome and bone marrow proliferative tumors is a major indication of allogeneic hematopoietic cell tumor. Allogeneic hematopoietic stem cell transplantation (HSCT) is the long-term option and the only radical treatment to save the life of SCN patients.^[27–29]^ It can cure cancers of the blood system and congenital and acquired diseases of the hematopoietic system.^[30]^ HSCT is currently the only treatment option for patients who do not respond to G-CSF and those who develop MDS/AML.^[31]^ The cases after the symptomatic treatment of G-CSF are still repeated infection. After the consultation of pediatric hematology, the patient underwent HSCT after comprehensive treatment of risks and benefits, and the therapeutic effect remains to be tracked. Hematopoietic stem cell transplantation includes conditioning with high-dose chemotherapy or radiotherapy to reduce bone marrow cells, followed by infusion of normal stem cells and follow-up after HSCT implantation. After HSCT, long-term immunosuppressants are required to prevent immune rejection.^[32]^ Reconstitution of the immune system in HSCT recipients takes at least several months. Reduced salivary flow and the use of topical corticosteroids and cyclosporine increase the risk of oral bacterial and viral infections and drug-induced gingival hyperplasia. Conditioning regimens such as chemotherapy and radiotherapy secondary to HSCT may lead to later oral changes such as xerostomia, dental caries, periodontal disease and dental dysplasia. Studies have shown that removal of oral infectious foci before HSCT conditioning can significantly reduce the incidence of oral complications.^[33]^ Most of the pediatric patients receiving hematopoietic stem cell transplantation are in the critical period of permanent tooth germ calcification development in alveolar bone. Therefore, potential alterations in craniofacial development and tooth eruption, general condition and medications being used should be considered in the treatment regimen adopted by the dentist for different age groups. Multidisciplinary team cooperation should be strengthened and comprehensive plans should be formulated to reduce the risk of infection in patients after transplantation. The transplantation team should encourage patients to have regular oral follow-up every 3 to 6 months as much as possible to prevent the occurrence of oral lesions such as dry mouth and periodontal disease.^[34]^

The child was admitted to the hospital due to oral manifestations such as gingival swelling and bleeding throughout the mouth. Periodontal disease in children is extremely rare in clinical practice. After multidisciplinary consultation, the child was diagnosed with SCN combined with periodontitis. This suggests that dentists should pay enough attention to children with periodontitis in clinical work, and carry out necessary physical examinations to screen for possible diseases. The occurrence and evolution of periodontal disease largely depends on the host immune response. The main goal of periodontal treatment for SCN patients should be the prevention of dental and periodontal infections through plaque control and the maintenance of periodontal health. In this case, the gingival inflammation symptoms were improved after periodontal treatment. Severe neutropenia leads to immune homeostasis disorder and increases the susceptibility of SCN patients to periodontal disease. However, the management of these patients’ oral health is considered to be complex. Patients with SCN are susceptible to infection due to their low immune function, so oral antibiotics should be used to prevent infection before starting periodontal treatment. Because the severity of bleeding is unpredictable, special precautions need to be taken before initiating any treatment. But while SCN is considered a hematologic disorder, spontaneous bleeding is actually uncommon when patients are treated orally. There was no significant spontaneous bleeding during treatment, including in this patient. Tozum et al reported that regular periodontal treatment and effective self-oral cleaning of SCN patients could improve their general condition and relieve the periodontitis symptoms of SCN children to a certain extent.^[11]^ In this case, there was no significant change in blood routine indexes before and after treatment, which may be due to the short follow-up time interval, which remains to be further explored. Therefore, SCN patients should be urged to maintain good daily oral hygiene. Once the diagnosis is made, individualized treatment plans can be developed for children according to the characteristics of the occurrence and development of the disease and its impact on oral health, which can effectively control the impact of periodontal disease and reduce the occurrence of serious infection.^[35]^

## Author contributions

**Conceptualization:** Zhu-Ling Guo, Min Yao.

**Data curation:** Min Yao, Si-Yu Tao.

**Formal analysis:** Min Yao, Si-Yu Tao, Zhu-Ling Guo.

**Funding acquisition:** Zhu-Ling Guo.

**Investigation:** Zhu-Ling Guo, Min Yao.

**Methodology:** Si-Yu Tao, Yu-Lei Dong, Xue-Jing Lin.

**Resources:** Min Yao, Zhu-Ling Guo.

**Software:** Si-Yu Tao, Yu-Lei Dong, Xue-Jing Lin.

**Supervision:** Min Yao, Zhu-Ling Guo.

**Validation:** Min Yao, Si-Yu Tao.

**Visualization:** Min Yao, Si-Yu Tao.

**Writing – original draft:** Si-Yu Tao, Min Yao, Diwas Sunchuri.

**Writing – review & editing:** Si-Yu Tao, Min Yao, Zhu-Ling Guo.
